# A 30 m vegetation map for the source regions of the Yangtze and Yellow Rivers from 2020–2025 ground surveys

**DOI:** 10.1038/s41597-026-07438-2

**Published:** 2026-05-14

**Authors:** Defu Zou, Xiaodong Wu, Guojie Hu, Tonghua Wu, Qiu Jin, Liangang Chen, Yanjun Bian, Cong Wang, Yuting Lan, Lin Zhao, Zhibin Li

**Affiliations:** 1 Cryosphere Research Station on Qinghai–Tibet Plateau, State Key Laboratory of Cryospheric Science and Frozen Soil Engineering, Northwest Institute of Eco–Environment and Resources, Chinese Academy of Sciences, Lanzhou, 730000 China; 2 Qinghai Haibei National Field Research Station of Alpine Grassland Ecosystem, Northwest Institute of Plateau Biology, Chinese Academy of Sciences, Xining, 810001 China; 3 State Key Laboratory of Hydrology–Water Resources and Hydraulic Engineering, Nanjing Hydraulic Research Institute, Nanjing, 210029 China; 4 School of Civil and Hydraulic Engineering, Lanzhou University of Technology, Lanzhou, 730050 China; 5 School of Geographical Sciences, Nanjing University of Information Science & Technology, Nanjing, 210044 China

## Abstract

An up-to-date vegetation map is essential for characterizing current ecosystem conditions in the source regions of the Yangtze and Yellow Rivers (SRYYR) under ongoing climate change. Here, we release a region-specific 30 m vegetation type dataset that represents typical conditions in the early 2020s. The map was created using systematic ground survey data from 1,168 georeferenced sites collected from 2020 to 2025. A random forest classifier was trained using Landsat imagery and environmental predictors to generate the vegetation type map. Sample representativeness was quantified by comparing predictor distributions between survey sites and the study area. The Euclidean distance ranged from 2.99% to 8.85% and Pearson’s r from 0.59 to 0.97. The final map had an overall accuracy of 79.56% and a Cohen’s kappa of 0.74. Unlike existing plateau-wide products, this dataset explicitly delineates alpine swamp meadows (ASMs) and improves the spatial representation of alpine shrublands (ASHs), providing an updated baseline for permafrost–vegetation coupling and related ecohydrological processes in the SRYYR.

## Background & Summary

The source regions of the Yangtze and Yellow Rivers (SRYYR), known as the Chinese Water Tower, are among China’s most important ecological and hydrological zones, supplying key water and ecosystem services to the downstream reaches of both basins^1,2^. Situated in the central area of the Qinghai–Tibet Plateau (QTP), the SRYYR supports representative alpine ecosystems with extensive permafrost, which cover >60% of the region and significantly influence soil thermal and moisture regimes, thereby regulating surface hydrological and ecological processes^3–5^. Vegetation types integrate these hydrothermal constraints and provide spatially explicit indicators of ecosystem states across the SRYYR. Type-resolved information is therefore essential for diagnosing permafrost–vegetation interactions and their implications for coupled carbon and water cycling^6–8^.

Over the recent decades, the spatiotemporal vegetation distribution in the SRYYR changed markedly under the combined influences of climate change and human activities. During the 1980s and 1990s, extensive alpine grasslands degraded due to overgrazing and declining soil moisture^9–11^. Since the early 2000s, satellite-derived vegetation indices have indicated a significant regional greening trend^12,13^, attributed to a warmer and wetter climate together with large-scale ecological restoration policies^14^. However, localized browning persists in high-elevation areas and in areas of degraded permafrost, where drought stress and limited soil water availability have reduced vegetation productivity^15–18^. These spatially contrasting vegetation trajectories are accompanied by vegetation type transitions, with expansions of some communities (e.g., low-cover grasslands) and contractions of others (e.g., wetlands and dense meadows)^13,19,20^. These vegetation dynamics highlight the need for an updated high-resolution vegetation map that depicts fine-scale vegetation patterns and class boundaries in the SRYYR.

At the QTP scale, land-cover and vegetation mapping have advanced substantially in terms of spatial resolution, temporal coverage, and mapping approaches. Recent efforts have produced fine-resolution (10 m) static maps that depict major vegetation types and their spatial patterns^21^, as well as Landsat-based 30 m datasets that provide consistent annual to decadal records of changes in vegetation cover from 1990 onward^22,23^. In parallel, mapping frameworks integrating spectral, topographic, and climatic predictors, together with plateau-wide training samples visually interpreted from satellite imagery, have improved the consistency of vegetation mapping across the plateau^24–27^. These datasets capture the broad distribution of major vegetation belts at the plateau scale. However, their thematic detail and spatial representation in the SRYYR, characterized by rugged terrain, patchy landscapes, and dense ecotones, are often insufficient for assessing ecologically important fine-scale heterogeneity.

These limitations partly reflect training data and modeling priorities in many plateau-wide products. Recent QTP-scale maps have used visually interpreted stable samples as training data^21–23^. Although this strategy ensures temporal consistency and high overall accuracy (OA) in plateau-wide applications, it is less reliable in heterogeneous mountain environments where mixed and transitional pixels are frequent. Ecotones are often simplified to maintain map consistency^28,29^. In practice, these samples are commonly obtained from spectrally homogeneous patches, resulting in an insufficient representation of rare and fine-grained classes in the training set^30^. Variable importance analyses have shown that despite the inclusion of spectral and topographic predictors, precipitation- and temperature-related variables often dominate model outcome^22,25^. Consequently, these products are characterized by smooth climate-driven gradients rather than by fine-scale patterns, particularly in small, hydrologically regulated vegetation units.

Two such vegetation types are alpine swamp meadow (ASM) and alpine shrubland (ASH), which are informative for ecosystem process studies in the SRYYR yet are frequently under-represented in existing products. ASM is often treated as a subtype of alpine meadows or wetlands in vegetation classification systems^31,32^ despite being functionally distinct. ASM is characterized by sustained surface waterlogging and high biomass, and is typically associated with organic-rich soils and ice-rich permafrost^33–36^. Accordingly, this vegetation type is widely used as a separate functional unit in permafrost and ecosystem studies^37–39^. The SRYYR has extensive areas of ASMs on the QTP; however, most existing QTP-scale products do not distinguish this class. The only permafrost-zone vegetation map^40^ depicting ASM has 1 km resolution, which is too coarse to resolve small wetland–meadow complexes. ASH occupies only a small proportion of the landscape. These areas are indicative of local hydrothermal and permafrost conditions. In the Yellow River headwaters, the upper shrubline at ~4200–4400 m a.s.l. closely coincides with the lower limit of widespread permafrost^41^, providing a proxy for transitions in the permafrost state and associated environmental regimes. ASH occurs primarily as small, topographically constrained patches on mountain slopes. This class is often included in grassland or wetland classes in QTP-wide products^22^, limiting the representation of its boundaries and ecological roles.

To improve the thematic representation and spatial resolution of ecologically important vegetation types in the SRYYR, we conducted a systematic ground-based vegetation survey from 2020 to 2025, comprising 1,168 georeferenced sites covering major vegetation types and their transition zones. Using these field observations as training data, we integrated multi-source remote-sensing imagery and environmental predictors within a random forest (RF) classification framework to produce a spatially continuous 30 m resolution vegetation map for the entire SRYYR. The dataset includes five vegetation types: ASM, ASH, alpine meadow (AM), alpine steppe (AS), and alpine desert (AD). Unlike existing QTP-level vegetation products, this region-specific dataset provides improved delineation of ASM and ASH and better captures fine-scale heterogeneity and vegetation boundaries typical of alpine landscapes in the SRYYR. The dataset provides a region-specific 30 m reference for analyses of permafrost–vegetation coupling, hydrological processes, and carbon cycling under ongoing climate change.

## Methods

### Study area

The SRYYR is located in the eastern QTP (90.55°–103.41°E and 32.16°–36.12°N), covering an area of ~2.7 × 10^5 ^km^2^ ^42^. The study area comprises the drainage basins upstream of the Zhimenda and Tangnaihai hydrological stations, representing the headwater regions of the Yangtze and Yellow Rivers, respectively. The terrain is characterized by rugged mountains interspersed with broad high-elevation plains and basins. Elevations are mostly >2600 m, with a mean altitude of 4464 m^43^. The region has an alpine continental climate with pronounced east–west hydrothermal gradients. The mean annual air temperature (MAAT) increases from approximately –5 °C in the west to 2 °C in the east, and mean annual precipitation (MAP) increases from ~300 mm to >600 mm, with >80% occurring from June to September^44,45^. Extensive permafrost areas and well-developed river networks regulate regional hydrological and ecological processes^46,47^. Vegetation distribution follows these gradients, transitioning from AD and AS in the colder, drier west to AM and ASM in the warmer, wetter east; ASH occurs as spatially patchy communities, primarily in the eastern SRYYR.

### Field survey data

**Fig. 1:**
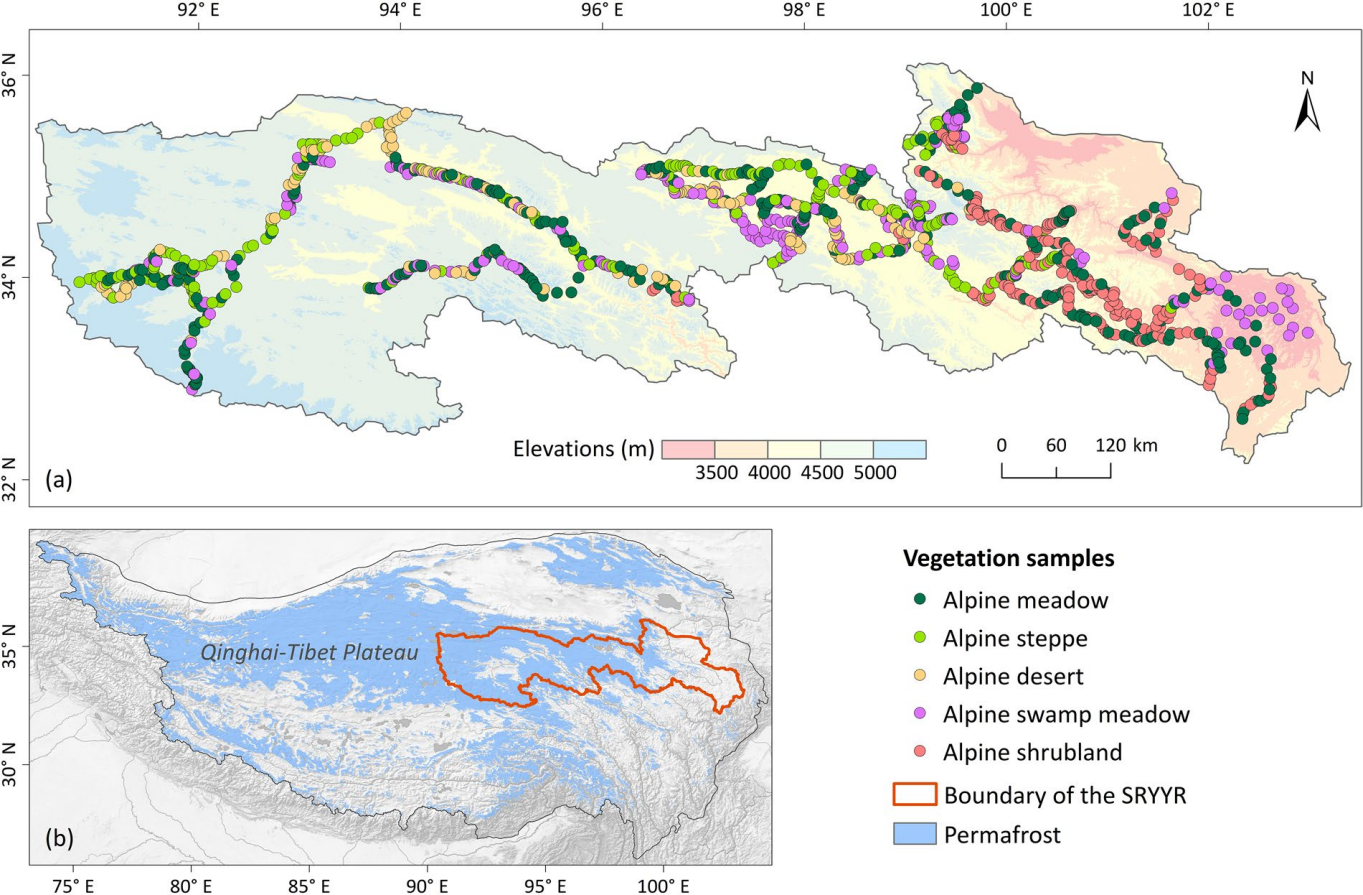
Study area and field sampling sites in the SRYYR. (**a**) Spatial distribution of the 1,168 vegetation survey sites. (**b**) Location of the SRYYR in the QTP.

Field surveys were conducted during 2020–2025 to characterize the spatial variability of natural vegetation across the SRYYR (Fig. 1). Sampling occurred along major transportation corridors (G109, G214, and S308) with targeted extensions toward headwater and tributary areas to improve coverage of key vegetation zones. Survey routes included the Tuotuo River basin (Yangtze River headwaters, 2020^48^), the Zhaling and Eling Lakes region (Yellow River headwaters, 2024), the central–eastern Yellow River source area (2025), and adjacent zones along the main highway transects.

**Fig. 2:**
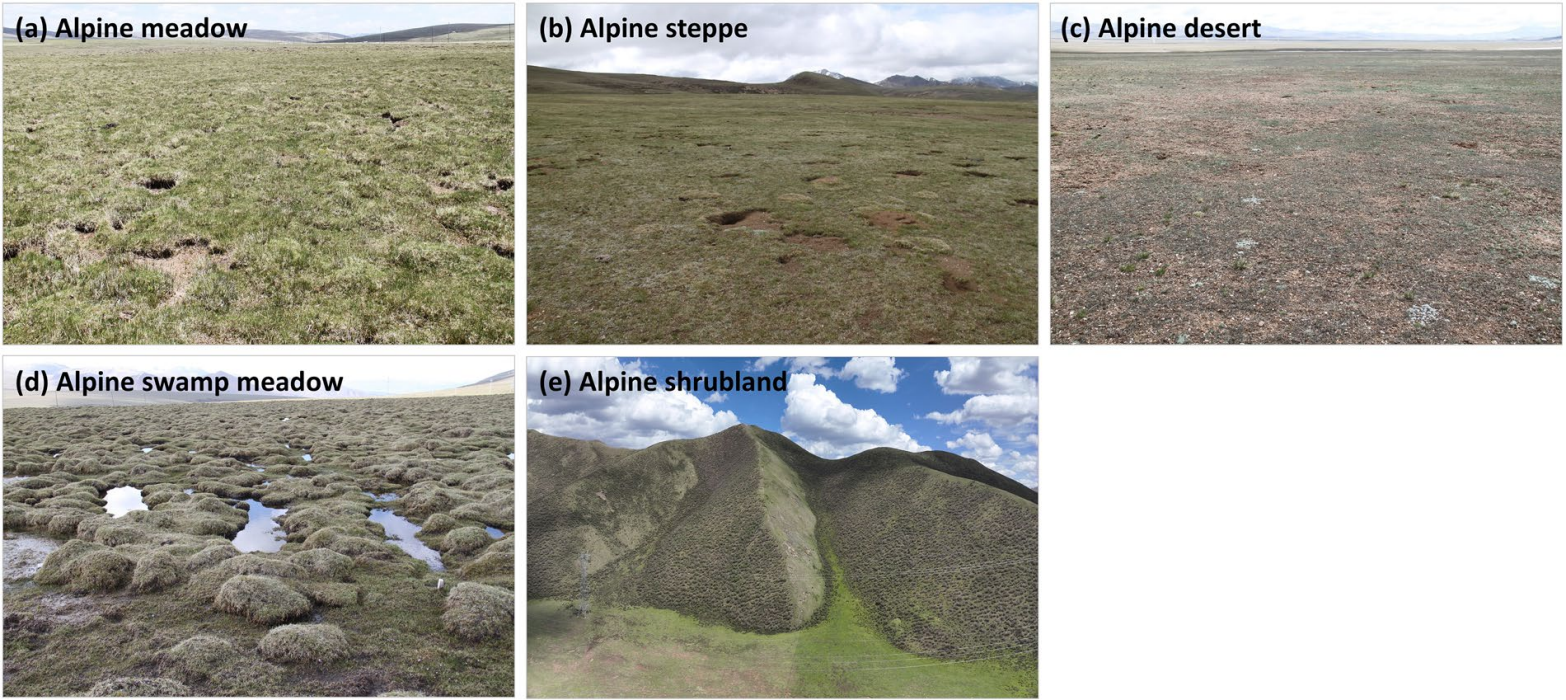
Representative field photographs of vegetation types in the SRYYR. (**a**) alpine meadow (AM), (**b**) alpine steppe (AS), (**c**) alpine desert (AD), (**d**) alpine swamp meadow (ASM), and (**e**) alpine shrubland (ASH).

A total of 1,168 sites were surveyed and assigned to five vegetation types: AM (313), AS (243), AD (156), ASM (235), and ASH (221). Sites were spaced at least 1 km apart to reduce spatial autocorrelation. These classes were assigned in the field based on vegetation composition, dominant life form, and habitat characteristics. Alpine grassland types (AM, AS, and AD) were identified based on species composition and dominant life forms following Wang *et al*.^40^. ASM was distinguished from AM by the presence of wet meadows and hydrologically constrained habitat, whereas shrubland communities dominated by *Salix oritrepha* and *Caragana jubata*^49^ were classified as ASH following the *Vegetation Map of China* (1:1,000,000)^50^. Representative field photographs are shown in Fig. 2.

Geographical coordinates and elevation were recorded using handheld GPS units (accuracy better than ±3 m). Field records underwent quality control, including removal of duplicate locations, consistency checks between vegetation descriptions and assigned type, and verification using high-resolution satellite imagery. The resulting field-labeled dataset was used as the reference for model training and validation of the 30 m vegetation map.

### Predictor variables

Predictor variables were selected to represent the main ecological controls on vegetation distribution in the SRYYR, including topographic setting, canopy spectral properties, surface moisture, and regional hydrothermal gradients.

#### Terrain factors

Topographic variables, including elevation (DEM), slope (SLP), and aspect (ASP), were derived from a 30-m resolution Shuttle Radar Topography Mission (SRTM) DEM. SLP and ASP were computed using the standard Horn algorithm, and a 3 × 3 moving mean filter was applied to reduce pixel-level noise. ASP was cosine transformed (ASP_cos) to represent north–south exposure. These terrain variables were used to characterize local topographic gradients relevant to vegetation distribution in the SRYYR.

#### Satellite imagery

Landsat 8 OLI surface reflectance data (Collection 2, Level-2; NASA/USGS) for 2020–2025 were accessed and processed through Google Earth Engine (GEE) to maintain a consistent sensor source across the compositing period. We limited observations to the growing season (June–September) and removed cloud- and shadow-contaminated pixels using the Level-2 quality-assurance layer to retain cloud-free samples. For each year, the remaining observations were aggregated as a growing-season mean, and annual means were summarized as a 2020–2025 median to represent typical vegetation conditions. The resulting median composite provided reflectance data for bands 2–7, which were used as predictors and to derive five vegetation indices (normalized difference vegetation index (NDVI), enhanced vegetation index (EVI), soil-adjusted vegetation index (SAVI), normalized difference water index (NDWI), and bare soil index (BI); Table 1).

**Table 1:** Predictor variables used for vegetation classification and their data sources.

Predictor	Unit	Data source	Resolution (m)
Elevation (DEM)	m	SRTM DEM	30
Slope (SLP)	°	SRTM DEM	30
Aspect cosine (ASP_cos)	—	SRTM DEM	30
Blue band (B2)	—	Landsat 8 OLI	30
Green band (B3)	—	Landsat 8 OLI	30
Red band (B4)	—	Landsat 8 OLI	30
Near-infrared band (B5)	—	Landsat 8 OLI	30
Shortwave-infrared band 1 (B6)	—	Landsat 8 OLI	30
Shortwave-infrared band 2 (B7)	—	Landsat 8 OLI	30
Normalized difference vegetation index (NDVI)	—	Landsat 8 OLI	30
Enhanced vegetation index (EVI)	—	Landsat 8 OLI	30
Soil-adjusted vegetation index (SAVI)	—	Landsat 8 OLI	30
Normalized difference water index (NDWI)	—	Landsat 8 OLI	30
Bare soil index (BI)	—	Landsat 8 OLI	30
Soil moisture (SM)	m^3^/m^3^	Li *et al*. 2025^52^	100
Mean annual air temperature (MAAT)	°C	Peng *et al*. 2019^51^	1000
Mean annual precipitation (MAP)	mm	Peng *et al*. 2019^51^	1000

#### Climate and soil data

MAAT and MAP were obtained from Peng *et al*.^51^ at 1 km resolution and resampled to the 30 m Landsat grid using nearest-neighbor resampling to ensure spatial alignment. Soil moisture (SM) was obtained from Li *et al*.^52^, which was derived from ascending and descending satellite overpasses during 2020–2023 at 100 m resolution. Ascending and descending estimates were averaged to reduce orbital bias, and the resulting SM layer was resampled to 30 m. These variables were included to represent broad climatic and soil-moisture gradients relevant to alpine vegetation distribution.

### Sample representativeness

**Fig. 3:**
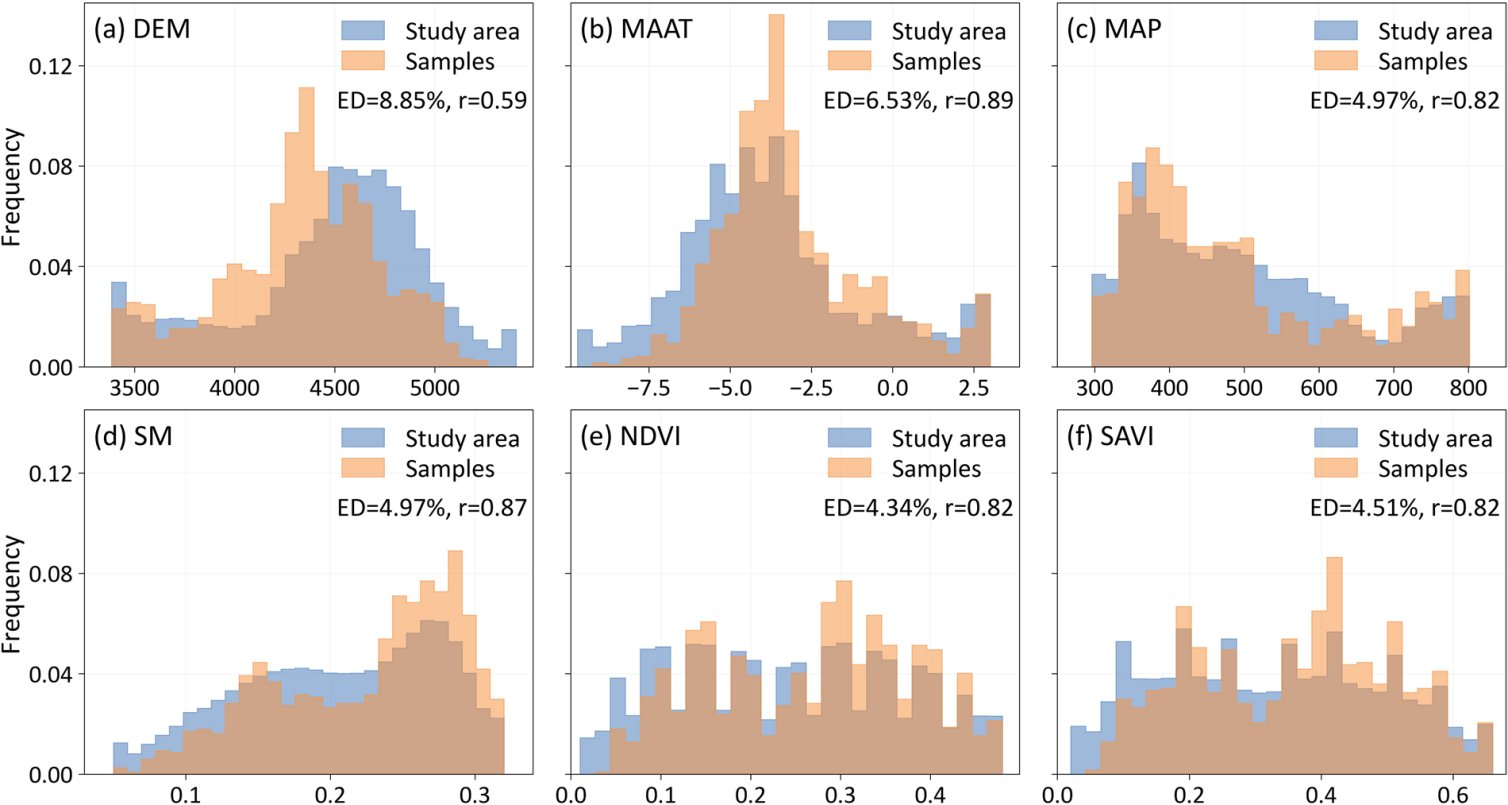
Sample representativeness for predictor variables. Normalized frequency histograms for (**a**) DEM, (**b**) MAAT, (**c**) MAP, (**d**) SM, (**e**) NDVI, and (**f**) SAVI. The Euclidean distance (ED) and Pearson correlation coefficient (r) were used to quantify similarity between the sample and study-area distributions.

The representativeness of the field samples was evaluated by comparing the frequency distributions of predictors between sample locations and the study area. For each variable, values from the sample locations and valid study area pixels were summarized as normalized frequency histograms using a 28-bin equal-width scheme. For variables with scaling factors, the same scaling was applied before comparison, and both distributions were clipped to the combined 1st–99th percentile range prior to binning. Distribution similarity was quantified using the Euclidean distance (ED) and the Pearson correlation coefficient (r) between the two probability histograms, with ED normalized and expressed as a percentage. Across all predictors, ED ranged from 2.99% to 8.85% (mean 5.44%; all ED <10%), while r ranged from 0.59 to 0.97 (mean 0.87), suggesting broadly comparable distributions between the samples and the study area. Representative comparisons for six variables are shown in Fig. 3. Elevation had a relatively large ED due to limited sampling sites at the highest elevations, whereas most other variables showed close correspondence between the two distributions. Overall, the samples provided broad coverage of the SRYYR predictor space.

### Vegetation mapping

**Fig. 4:**
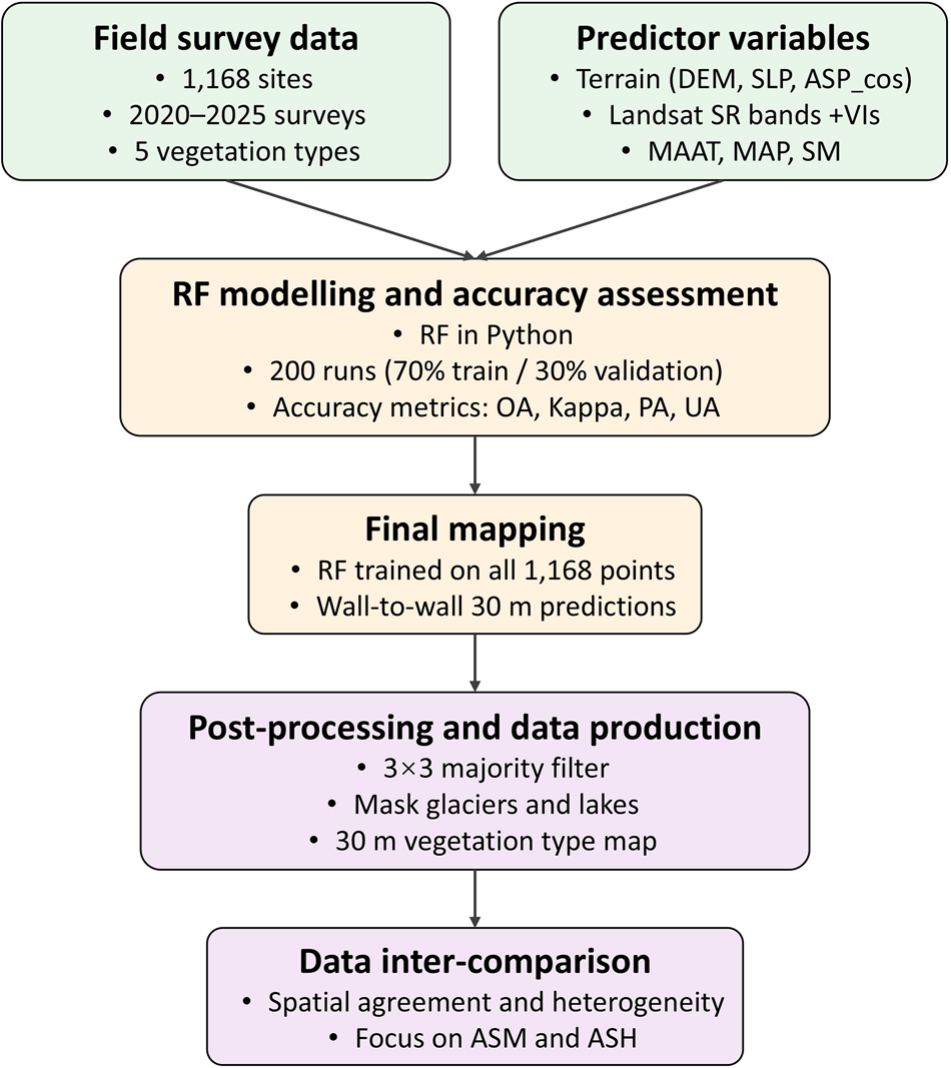
Workflow for generating the 30 m vegetation map for the SRYYR.

The mapping workflow is summarized in Fig. 4. All predictor layers were resampled to 30 m and projected to a common coordinate system to ensure pixel-wise alignment. Predictor values were extracted at the 1,168 quality-controlled field sites to build the training dataset for classification. An RF classifier was used because it performs well with high-dimensional predictors and has been widely applied to vegetation mapping on the QTP^21–23,25,48^. The multi-class RF classifier was implemented in Python and configured with *n_estimators* = 1000 and *max_features* = 4. These hyperparameters were selected through preliminary tuning to balance classification accuracy and computational efficiency. A fixed random seed was used to ensure reproducibility, and all other RF hyperparameters were left at their default settings. No additional class weighting, re-sampling, or threshold adjustment was applied in the final model. The classifier was trained using the field-assigned labels for five vegetation types (AM, AS, AD, ASM, and ASH) and applied to the full predictor stack to extrapolate these classes across the SRYYR and obtain 30-m-resolution predictions.

### Accuracy assessment

The thematic accuracy of the vegetation map was assessed using Monte Carlo repeated random sub-sampling validation. In each of the 200 iterations, labeled sites were stratified by vegetation type and randomly split into calibration (70%) and independent validation (30%) subsets, thereby preserving class proportions and ensuring reproducibility across repeated validation runs. The RF model used the same hyperparameter settings as in the final mapping. It was trained on the calibration subset and applied to the validation subset. A confusion matrix was generated for each iteration to compute OA, Cohen’s kappa coefficient, and class-specific producer’s accuracy (PA) and user’s accuracy (UA). Metrics were summarized across iterations as mean ± standard deviation to characterize performance and its variability. Accuracy results are reported in the Technical Validation section.

### Post-processing

A 3 × 3 majority filter was applied to the initial RF classification to reduce salt-and-pepper noise, producing a more spatially coherent vegetation map. The wall-to-wall predictions were masked to exclude non-vegetated surfaces. Glacier areas were masked using the Second Glacier Inventory of China^53^ (v1.0, 2006–2011; National Cryosphere Desert Data Center; accessed 20 Oct 2025), and lake areas were masked using the China Lake dataset^54^ (1960s–2020; National Tibetan Plateau Data Center; accessed 12 Oct 2025).

## Data Records

**Fig. 5:**
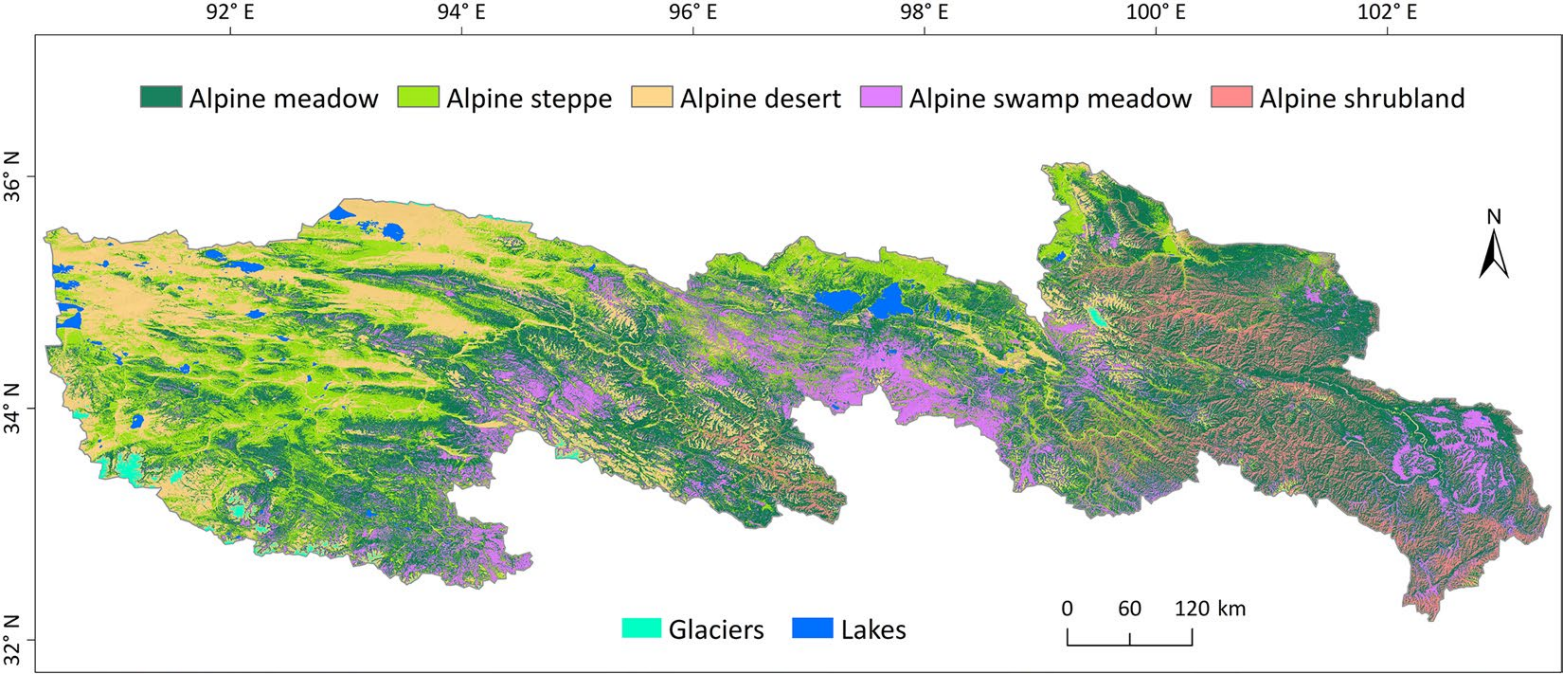
The predicted 30 m vegetation type map of the SRYYR.

The vegetation type dataset for the SRYYR is publicly available at the National Tibetan Plateau/Third Pole Environment Data Center^55^. The dataset is provided as a single GeoTIFF raster (SRYYR_vegetation_map_30m.tif) at 30 m spatial resolution. Pixel values are stored as integers and encode five vegetation types: 1 = alpine meadow (AM), 2 = alpine steppe (AS), 3 = alpine desert (AD), 4 = alpine swamp meadow (ASM), and 5 = alpine shrubland (ASH) (Fig. 5). Glacier and lake areas were masked during post-processing, stored as NoData in the final raster, and excluded from class area-fraction calculations. Across the mapped area, AM is the dominant class (40.0%), followed by AS (20.7%) and AD (18.0%). ASM accounts for 13.4% and is concentrated in low-lying basins and valley floors in the central–eastern SRYYR, while ASH (7.8%) occurs mainly as scattered communities on mountain slopes in the eastern SRYYR.

## Technical Validation

### Classification accuracy

**Fig. 6:**
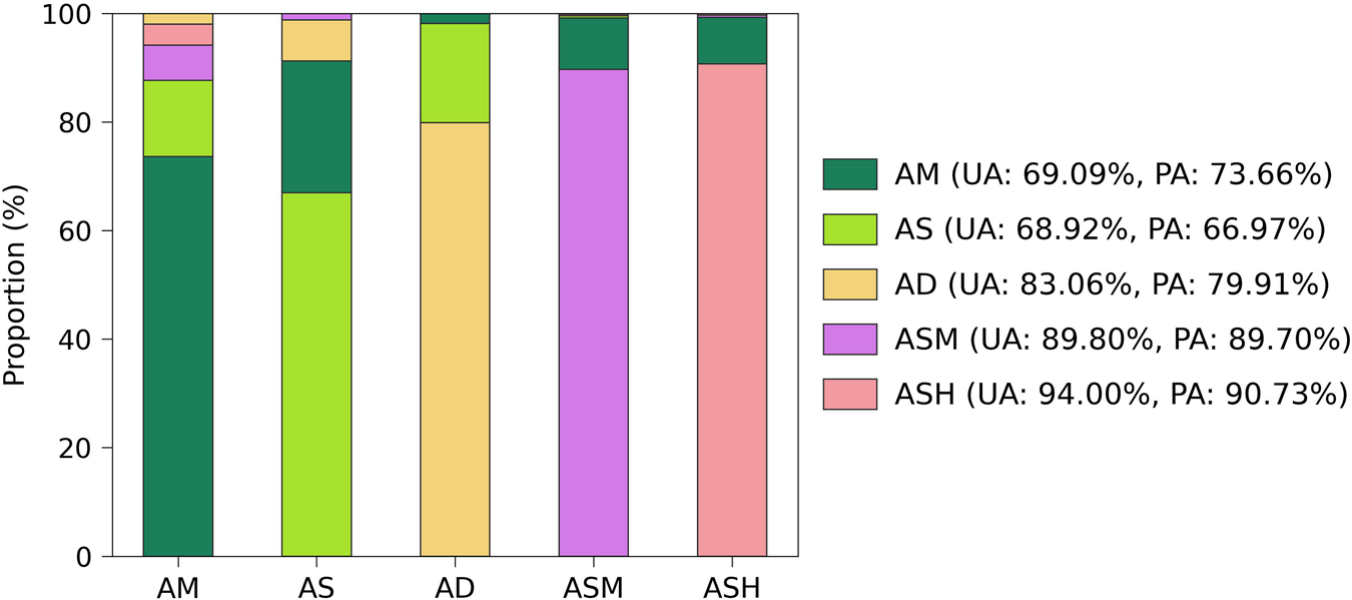
User’s and producer’s accuracies for five vegetation classes derived from Monte Carlo validation.

Across 200 independent runs, the mean OA was 79.56% ± 1.81%, and Cohen’s kappa was 0.74 ± 0.02, indicating substantial agreement. Class-specific accuracies are reported as PA and UA. ASH and ASM achieved the highest accuracies (PA/UA = 90.73%/94.00% and 89.70%/89.80%), followed by AD (79.91%/83.06%) (Fig. 6). AM and AS had lower accuracies (73.66%/69.09% and 66.97%/68.92%), consistent with broader within-class variability and gradual transitions among alpine grassland communities. Misclassifications occurred primarily between AM–AS and AS–AD, whereas ASH and ASM exhibited lower confusion than other classes.

### Comparison with previous products

**Fig. 7:**
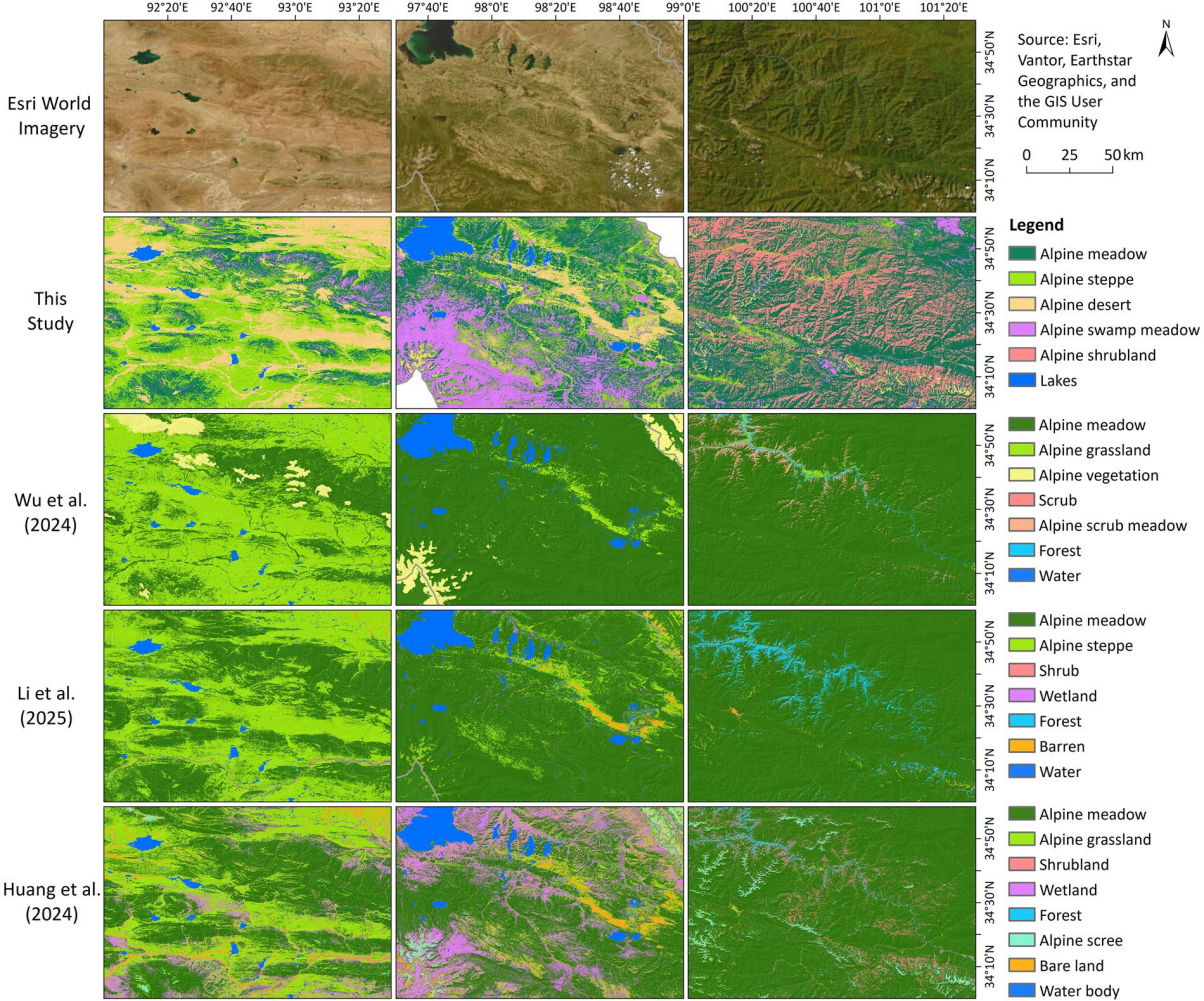
Spatial comparisons with three QTP-wide products in three sub-regions. Panels show our map and the 2020 (Wu *et al*.^22^), 2023 (Li *et al*.^52^), and 2022 (Huang *et al*.^21^) products using their original legends.

We conducted a qualitative spatial comparison with three recent QTP-wide products: the 2020 vegetation map produced by Wu *et al*.^22^, the 2023 land-cover map of Li *et al*.^23^, and the 2022 land-cover map of Huang *et al*.^21^, at comparable spatial resolutions (10–30 m). Because the classification schemes differ across products, particularly for ASM and ASH, pixel-wise quantitative comparisons based on the original legends would be difficult to interpret and potentially misleading. We therefore retained each product’s original legend for a transparent qualitative comparison. Three representative sub-regions at 34–35°N were selected to cover major west–east vegetation transitions (a dry western transition zone at ~93°E, the Eling Lake region at ~98°E, and a wetter, more heterogeneous eastern sector at ~101°E; Fig. 7).

At the regional scale, all products captured the broad hydrothermal gradient across the SRYYR, with steppe/desert classes dominant in the drier west and meadow/wetland classes increasing toward the wetter east. Differences were most evident in fine-scale heterogeneous landscapes. Our SRYYR map explicitly delineates ASM as a separate class in low-lying basins, whereas QTP-wide products represent these areas as AM or wetland depending on their legend definitions. Similarly, ASH is more spatially coherent in our map, with patches occurring primarily on mountain slopes and mid-elevation terrain, while the comparison products frequently merged shrub-dominated areas into broader grassland or mixed classes. Overall, the comparison suggests that the region-specific mapping strategy and dense field sampling improved the depiction of small-scale heterogeneity and vegetation boundaries in the SRYYR, particularly for ASM and ASH.

### Importance of input features

**Fig. 8:**
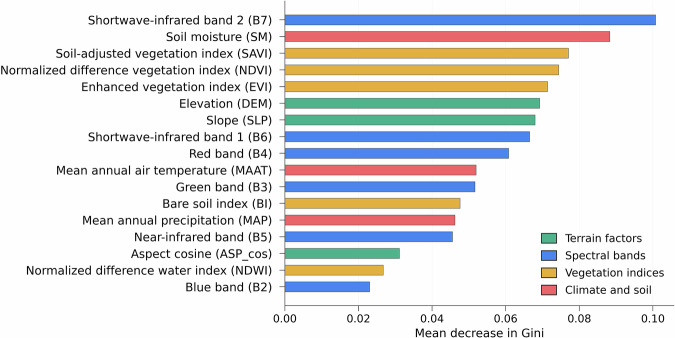
Relative importance of predictor variables in the final RF model, quantified by the mean decrease in the Gini index.

Predictor importance was evaluated using the mean decrease in the Gini index from the final RF model (Fig. 8). Spectral predictors from the Landsat composite ranked highest, with the shortwave-infrared band (B7) contributing most, followed by SM and vegetation indices (e.g., SAVI, NDVI, EVI). This ranking is consistent with the sensitivity of shortwave-infrared bands and vegetation indices to vegetation water content, canopy structure, and soil background, while SM provides complementary constraints on hydrological controls. Topographic variables (DEM and SLP) showed intermediate importance, reflecting terrain-driven differentiation of vegetation zones, whereas climate variables (MAAT and MAP) played a supplementary role relative to spectral predictors. These results suggest that Landsat-based fine-scale spectral information provides the most meaningful information for vegetation mapping in the SRYYR, whereas SM provides an important independent hydrological constraint, and topographic and climatic predictors contribute to a broader environmental context across local to regional scales.

### Limitations and prospects

Classification uncertainty is expected to be higher near vegetation boundaries and in very small heterogeneous patches, including AS–AD transitions in the arid western SRYYR, ASM–AM transitions in low-lying basins, and ASH–AM transitions on mountain slopes in the eastern SRYYR. In these areas, gradual changes in community composition and SM conditions are difficult to capture with discrete classes at 30 m resolution. Mixed pixels can increase confusion among spectrally similar types, and local vegetation boundaries may shift interannually. Although survey routes were extended beyond major corridors and sites were spaced > 1 km apart, field plots were still concentrated along accessible transects, and some remote areas were sparsely sampled. In addition, pixel-wise quantitative inter-comparison with existing QTP-wide products was constrained by differences in classification systems, particularly for ASM and ASH, which were not included as separate classes in the comparison products. For applications focusing on local ecotones or very small patches, we recommend combining the map with high-resolution imagery and site knowledge.

## Usage Notes

This dataset provides a region-specific 30 m vegetation baseline for the SRYYR, designed for regional to basin-scale analyses of alpine ecosystem patterns and their links to hydrothermal and permafrost controls. The map represents typical early-2020s vegetation conditions based on multi-year field surveys (2020–2025) and a multi-year Landsat growing-season composite spanning the same period. It should therefore be interpreted as a multi-year regional baseline rather than a year-specific map, and users should avoid using it alone for year-to-year change detection.

When this dataset is integrated with QTP-wide land-cover/vegetation products, pixel-wise quantitative comparisons may be misleading because the class legends were not identical (e.g., ASM and ASH were part of the meadow/wetland or grassland classes in other products). For cross-product synthesis, we recommend developing an explicit class crosswalk (e.g., aggregating to broader meadow/steppe/desert/shrubland groups) before statistical or quantitative comparison. For local applications that focus on class boundaries or very small vegetation patches, users may combine the 30 m map with high-resolution imagery and site knowledge, and consult the “Limitations and prospects” section for areas with higher uncertainty.

## Data Availability

The vegetation type dataset for the SRYYR is publicly available at the National Tibetan Plateau/Third Pole Environment Data Center 10.11888/Terre.tpdc.303202. On the landing page, click “Download” to generate temporary FTP account credentials for anonymous download (no registration required).

## Code availability

The scripts used to generate the Landsat composites in GEE, to train the RF classifier and produce the wall-to-wall vegetation map, and to perform Monte Carlo validation and accuracy reporting (Python) are available from the National Tibetan Plateau/Third Pole Environment Data Center 10.11888/Terre.tpdc.303202^55^.
